# Supplementary material for: Chromothripsis during telomere crisis is independent of NHEJ, and consistent with a replicative origin
Source: Genome Res. 2019 May;29(5):737–49. doi: 10.1101/gr.240705.118 (PMC6499312; doi:10.1101/gr.240705.118)
Supplement: Supplemental Material [file supp_gr.240705.118_Supplemental_file_1.zip › contigs/annotated_contigs/DB79/contig.2.DB79_length_682_mean_cov_7.3431085044.docx]

**DB79_length_682_mean_cov_7.3431085044**

```
TCTCATTATGTATTATATTATACAGTTTCTAACCTTAAAGATTATTATCCTTCTTTGACAGGTTTGCACAGTGTACTGAATGATTAAGG
 >chr11:37074420-37074774 + E=3e-195
TAGGTGCTTTCTTATTTTCCTGTCAAGAACATTTATATATCTGGCTACAAAAGTGATATAGGATTACTGACAAAGTTAAAACACACAGA

GGGATACAAGACAAAAAACAAAATTGCTTACAACTTTACAAGTCAAAATAACTGTGGCTAACATTTTAGTGCATAACTTTAGTGCATTG

TTTTTAAAATGACTTTTGTTTTCTTCCATGACCTCTGGGAAGGCCGTTAACTATATATATATATATATATATATATATATATATAT|A|
                                                                                       >
GCAGGATTCTGTATATGCAGTGCCTAGCACATAGCAGCTACGTCATTAATGTTTGCTGAATGGATCAATAAAAAACCTGAGAGGAGGAA
chr11:61357190-61357519 - E=3e-186
GATAAAGATGGATAGTAAACGGGCGATCCTTGCCCAGAAAGAGCTCCCAGTCCATTGCAGGGGTGGAGGAGGTGGACAGAAAGCAGGAT

TGAAGAGCTGTATAAATGCTATCAGCGTAGACAACTCCATCACCGAAATGCTGAGAAGTGCCATGGGGCTACAGAAGAGGAGCATTCAG

ACTCACCTGGGAGAATCAGGGATGGCTCCCTGGACAAAGTGACAGTGAAGATGGGCTTTGA
```
